# One Pot Synthesis of Copper Oxide Nanoparticles for Efficient Antibacterial Activity

**DOI:** 10.3390/ma16010217

**Published:** 2022-12-26

**Authors:** Rajaram Rajamohan, Chaitany Jayprakash Raorane, Seong-Cheol Kim, Yong Rok Lee

**Affiliations:** School of Chemical Engineering, Yeungnam University, Gyeongson 38541, Republic of Korea

**Keywords:** microwave-assisted synthesis, CuO, XPS analysis, BET, antimicrobial activity, biofilm

## Abstract

The unique semiconductor and optical properties of copper oxides have attracted researchers for decades. However, using fruit waste materials such as peels to synthesize the nanoparticles of copper oxide (CuO NPs) has been rarely described in literature reviews. The main purpose of this part of the research was to report on the CuO NPs with the help of apple peel extract under microwave irradiation. Metal salts and extracts were irradiated at 540 W for 5 min in a microwave in a 1:2 ratio. The crystallinity of the NPs was confirmed by the XRD patterns and the crystallite size of the NPs was found to be 41.6 nm. Elemental mapping of NPs showed homogeneous distributions of Cu and O. The NPs were found to contain Cu and O by EDX and XPS analysis. In a test involving two human pathogenic microbes, NPs showed antibacterial activity and the results revealed that the zone of inhibition grew significantly with respect to the concentration of CuO NPs. In a biofilm, more specifically, NPs at 25.0 µg/mL reduced mean thickness and biomass values of *S. aureus* and *E. coli* biofilms by >85.0 and 65.0%, respectively, with respect to untreated controls. In addition, environmentally benign materials offer a number of benefits for pharmaceuticals and other biomedical applications as they are eco-friendly and compatible.

## 1. Introduction

Infections caused by bacteria are a growing public health concern and are the major reason for the spread of serious diseases worldwide, with millions of new cases and deaths per year [1,2]. Fresh products contaminated with bacteria are the most common source of bacterial illnesses. Therefore, individual consumers, industries, and regulatory authorities are concerned about food safety. Most commonly, *Salmonella*, *Escherichia coli*, and *Staphylococcus aureus* cause foodborne illnesses [2,3]. It is possible for people to experience diarrhea, abdominal cramps, and nausea after consuming food that contains these pathogenic bacteria. As a result, it may also cause chronic illnesses such as cancer, brain disorders, kidney failure, and liver failure [4]. Bacterial infections caused by these microorganisms remain a challenge because they often form biofilms on surfaces and have developed an enhanced resistance to commonly used antimicrobial agents [5]. Drying, pickling, thermal processing, and freezing are traditional methods of extending food shelf life. As a result of these procedures, most nutrients in food are denaturalized or destroyed [6].

Metal oxides are widely used in modern technology owing to their excellent electrical, chemical, and optical properties [7,8]. Furthermore, metal oxide nanoparticles are increasingly being investigated for their biological properties. Several researchers have assessed the biologically effective activity of nanoparticles of metal oxide, especially copper oxide (CuO), which have established improved biological activity in comparison with metal NPs [9]. Among the various metal and their oxide-based NPs, copper has gained recognition due to its high redox potential [10]. Copper-based NPs such as CuO, amorphous and crystalline CuS, CuPO_4_, and CuI is reported to have biological activity [11,12,13,14,15].

A variety of physical and chemical routes have been used to obtain CuO NPs with desired morphologies [16,17,18,19,20]. It is important to note, however, that these methods require a lot of labor, a lot of energy, an intensive route, and hazardous chemicals [21]. It is, therefore, essential that new biocompatible approaches be developed that can help to rectify the above-mentioned limitations [22]. The synthesis of NPs including metal as well as metal oxide is shifting from physical and chemical methods to biological methods termed biosynthesis or green synthesis [23,24]. Recently, fruit peels have been used to synthesize metal or metal oxide nanoparticles [25,26,27]. Due to its sustainability, cost-effectiveness, and simplicity, the photosynthesis of CuO NPs has gained more attention recently [28]. The purpose of this research work was to provide an environmentally friendly synthetic process for CuO NPs characterization followed by an antibacterial activity.

To the best of our knowledge, there are no other reports on the synthesis of CuO NPs with the help of apple peel extracts via microwave irradiation. The obtained NPs were characterized by analytical techniques which include XRD, FE-SEM, HR-TEM, XPS, and BET surface analysis. Moreover, our aim from the application point of view was to test the antimicrobial and antibiofilm efficacy of synthesized CuO NPs against Gram-positive as well as Gram-negative bacterial pathogens.

## 2. Materials and Methods

### 2.1. Materials

The Sigma Aldrich Company, Seoul, Republic of Korea, provided copper nitrate (molecular formula: CuNO_3_·2H_2_O, Purity: >99%) for the synthesis of NPs without further purification. The CuNO_3_·2H_2_O was readily soluble in distilled water. After thoroughly washing the glassware with distilled water, they were dried in the oven for 30.0 min to avoid contamination of the glassware by the deposition of impurities.

### 2.2. Preparation of Apple Peel Extract

From the fresh and delicious apples, apple peel extract has been prepared with the help of a homogenizer. A detailed procedure has been given in the Supplementary Materials.

### 2.3. Preparation of CuO NPs

**Scheme 1:**
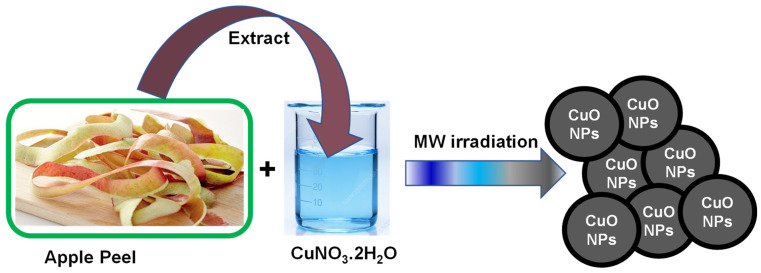
Synthesis of bio-reduced CuO NPs.

AP extract (10.0 mL) and CuNO_3_·2H_2_O (3.146 g in 50.0 mL distilled water) were taken separately with the required amounts. A scheme for synthesizing NPs is shown in Scheme 1. An extract of AP was typically added directly to aqueous solutions of CuNO_3_·2H_2_O at 0.003 mol/L with constant stirring for 10 min at 60.0 °C [29]. A pale blue color developed in the solution after ten minutes. In a microwave oven (Panasonic N-ST342, Seoul, Republic of Korea), the mixture was irradiated for 5 min at 90.0 °C under an N_2_ atmosphere. Pale blue turned into light brown in less than a minute. A 15 min centrifuge with three 5 min intervals was used to separate the CuO NPs from the solvent, followed by numerous washes with ethanol and also deionized water. Centrifugation caused the CuO NPs to sediment after washing and was followed by sonication in water for one minute. A refrigerator was used to store the CuO NPs after they were dried for 24 h at 400.0 °C. It was highly probable that CuO nanoparticles would form NPs when exposed to air. AP extract was an efficient reducing agent as well as a stabilizing agent in the formation of NPs.

### 2.4. Antibacterial Activity

#### 2.4.1. In-Vitro Antibacterial Efficacy

The assessment of the antibacterial efficacy of CuO NPs was performed using the agar well diffusion method [30]. For this study, *E. coli* as Gram-negative bacteria (ATCC 43895) and *S. aureus* as Gram-positive bacteria (ATCC 6538) were used. Briefly, on sterile Mueller Hinton agar (MHA) plates, overnight cultures of each bacterial strain at 0.5 McFarland standard were spread, which were pierced with a 7 mm diameter cork borer and loaded up with 50.0 μL of CuO NPs diluted in 1.0% DMSO at different concentrations (10.0 μg/mL, 200.0 μg/mL, and 300.0 μg/mL (*w*/*v*)). After the incubation process, the radius of the inhibition zone was measured by the use of a Vernier caliper. Clinical Laboratory Standards Institute (CLSI) bacteria and Cation-adjusted Mueller–Hinton broth media were used in this study. For reliable findings and reproducibility, experiments were carried out using at least two different cultures.

#### 2.4.2. Antibiofilm Potency of CuO NPs against Bacterial Pathogens

A biofilm experiment was carried out on 96-well microtiter plates using the crystal violet staining technique [31]. The initial turbidities of OD 0.05 (~10^6^ CFU mL^−1^) for *S. aureus* and OD 0.1 (~10^6^ CFU mL^−1^) for *E. coli* at 600.0 nm were inoculated into an LB culture media (final volume 300.0 μL) with or without the CuO NPs and incubated for 24 h without shaking at 37.0 °C. The formation of biofilm was confirmed by staining with 0.1% crystal violet for 30 min and washed frequently with distilled water and then 95.0% ethanol was added to each well. The absorbance of each plate well was recorded at 570.0 nm using a spectramax 190 microplate reader equipped with a xenon flash lamp (Molecular device, San Jose, CA, USA). Biofilm assays were carried out with two independent cultures in triplicate.

#### 2.4.3. Antibiofilm Potency of CuO NPs against Bacterial Pathogens

A biofilm observation of the CuO NPs against both Gram-positive and Gram-negative bacterial pathogens have been measured by CLSM, and the detailed procedure has been provided in the Supplementary Materials.

### 2.5. Instruments Used

The bio-reduced CuO NPs are characterized by analytical instruments, and details are provided in the Supplementary Materials.

## 3. Results and Discussion

### 3.1. Analysis of Bio-Reduced CuO NPs by DRS

**Figure 1:**
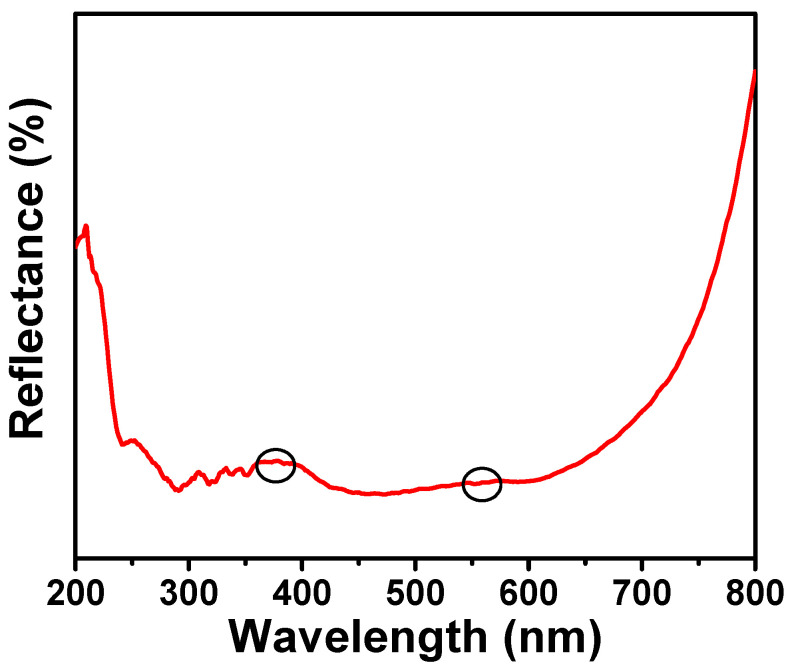
DRS spectra of bio-reduced CuO NPs.

Figure 1 shows the DRS spectrum of pure CuO NPs. DRS curves were measured by UV-VIS-NIR spectrophotometers. NPs exhibit absorption bands in the range of 365.0 nm [32]. At 560.0 nm, a peak was observed as a shoulder, which implies the existence of CuO on the surface of NPs [32]. Furthermore, there was a weak reflectance in both the UV and visible ranges (200.0–800.0 nm). It also provided information about the greater absorption in the regions, as it gives weak transmittance. The band gap for the NPs was also calculated from the spectra and the value was 1.58 eV. The bandgap energy was higher than the bulk CuO material [33] and very close to the synthesized CuO NPs [34,35]. In the results, it consists only of CuO NPs and not of Cu.

### 3.2. Analysis of Bio-Reduced CuO NPs by FT-IR Spectrum

**Figure 2:**
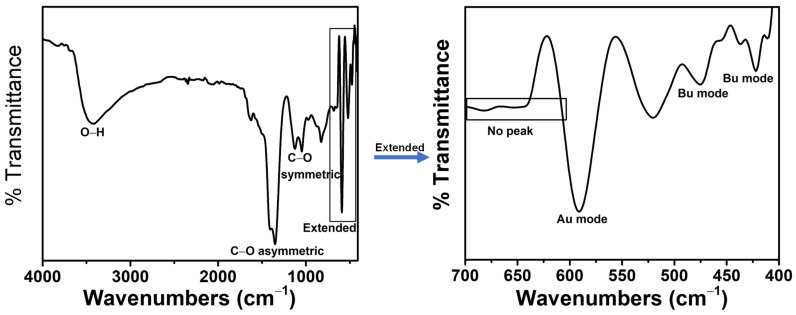
FT-IR spectrum of bio-reduced CuO NPs.

The effects of the peel extract used in the synthesis of NPs were analyzed by FT-IR analysis to obtain the structural and chemical properties of the synthesized metal oxides [36,37]. FT-IR spectra were recorded between 400.0 and 4000.0 cm^−1^ (Figure 2). CuO NPs have a peak at 3416.0 cm^−1^, which was correlated with hydroxyl group stretching. NPs have a peak at 1650 cm^−1^ caused by a bending O–H. Another peak at 1376.0 cm^−1^ was related to C-O asymmetry in NPs. Another peak in the structure was at 1115.0 cm^−1^, which was related to C-O symmetry. A peak at 533.0 cm^−1^ was associated with Cu-O bonds. There were three characteristic bands of CuO including the A_u_ mode, and two B_u_ modes of CuO appeared at 432.3 cm^−1^, 497.0 cm^−1^, and 603.3 cm^−1^, respectively [38]. The high-frequency mode can be observed at 603.3 cm^−1^ and it has been attributed to the Cu-O stretching vibration in the [101] direction. The [101] direction of the Cu-O stretching vibration has been linked to the other peak, which can be seen at 497.0 cm^−1^ [39]. Therefore, the FT-IR analysis indicates that CuO NPs are in their pure phase and have a monoclinic structure.

### 3.3. Analysis of Bio-Reduced CuO NPs by Raman Spectrum

**Figure 3:**
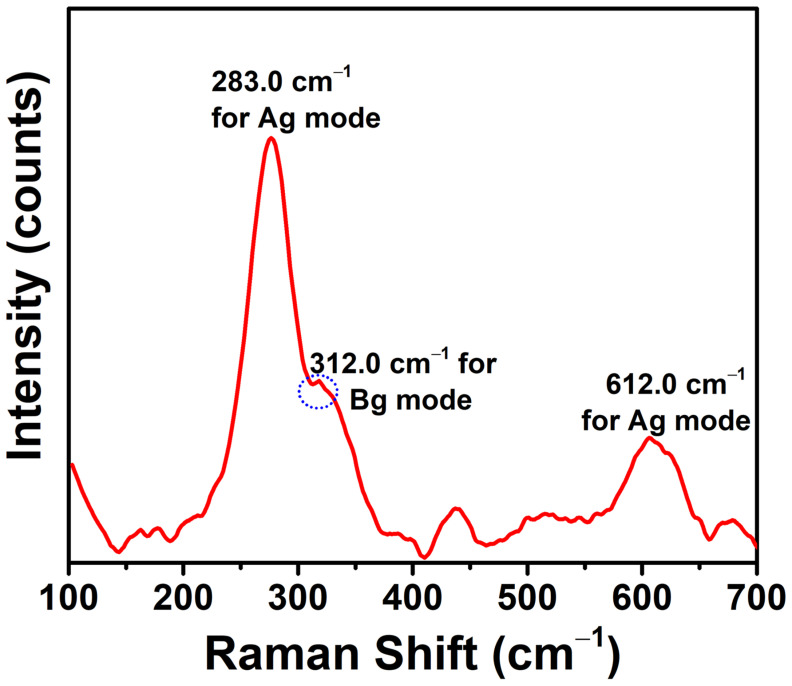
Raman spectra of bio-reduced CuO NPs.

Raman spectral analysis can be used as a major analytical technique to identify the vibrations of metal oxide NPs and local atomic arrangements and analyze their structural features [40,41]. It can also be used to determine how crystalline the NPs are. Figure 3 shows a strong peak at 283.0 cm^−1^, which is associated with the A_g_ mode of vibration. The weak peaks that appeared at 312.0 cm^−1^ and 612.0 cm^−1^ corresponded to the B_g_ modes of vibration [41,42]. Only vertically and with a displacement do oxygen atoms move to the b-axis in Raman modes for both the A_g_ and B_g_ bands. Decreasing the size of the NPs altered a Raman shift and bandwidth [42].

### 3.4. Analysis of Bio-Reduced CuO NPs by XRD

**Figure 4:**
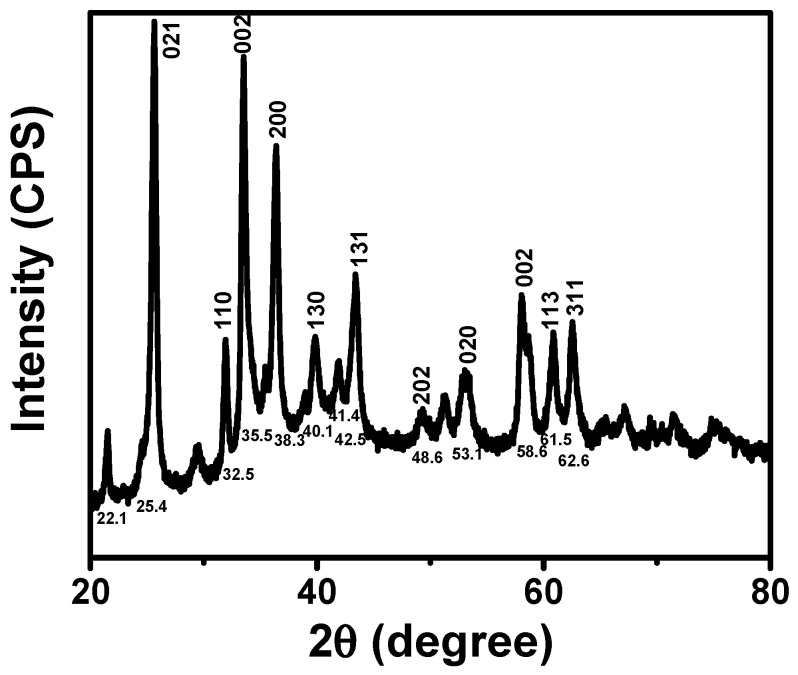
XRD patterns of bio-reduced CuO NPs.

Analysis of NPs obtained from metal oxides by XRD patterns is a powerful analytical technique to obtain information about the crystalline peaks [43]. Figure 4 shows the XRD patterns of CuO NPs at 2θ ranges from 20 to 80 (in degrees). According to the Joint Committee on Powder Diffraction Standards (JCPDS) database, crystalline phases were recognized. The patterns of NPs exhibited a significant peak at (2θ) 25.41, 32.51, 35.52, 38.32, 40.10, 42.51, 48.62, 53.10, 58.68, 61.52, and 62.64 (JCPDS01-080-1268) which belong to miller indices [021], [110], [002], [200], [130], [131], [202], [020], [002], [113], and [311], respectively [44,45]. Furthermore, there were some low-intensity peaks which may be due to the negligible amount of impurities in the NPs. Slight variations were observed in the obtained peaks at their position with respect to the JCPDS data, which may be due to the slight modifications in terms of phase on the surfaces of NPs. A strong intensity peak at 35.52 and a low-intensity peak at 38.32 appeared which revealed the formation of CuO and existed as the monoclinic phase [46]. Additionally, the sharp XRD patterns were evident for the crystalline nature as well as the monoclinic phase of the CuO on the whole surface of NPs. The lattice parameters a, b, and c were found to be 4.68 Å, 3.41 Å, and 5.08 Å, respectively. The average crystalline size was found to be 41.6 nm for the NPs according to the well-known Scherrer equation as follows [46,47],

D = (Kλ)/(β·cosθ)(1)

where D stands for crystallite size (nm); K stands for Scherrer’s constant that is associated with crystallite shape, normally taken as 0.9; β is the full width half maximum (radians), λ is the wavelength of the Cu Kα radiation (1.54 Å); and θ is the Bragg angle (Å).

### 3.5. Analysis of Bio-Reduced CuO NPs by FE-SEM

**Figure 5:**
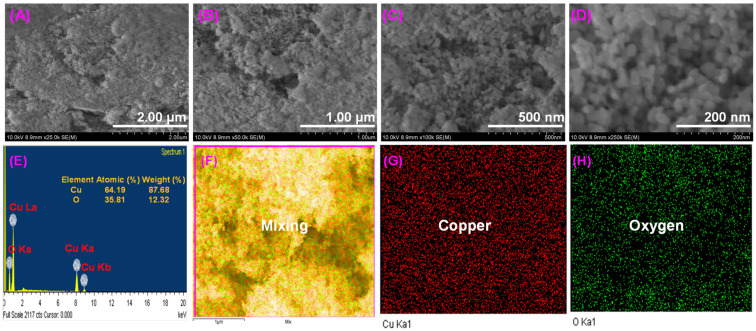
FE-SEM images of bio-reduced CuO NPs (**A**–**D**), EDX spectrum of bio-reduced CuO NPs (**E**), elemental mapping with mixing (**F**), elemental mapping of Cu (**G**), and elemental mapping of O (**H**).

FE-SEM images were used for visual examination and analysis of the surface morphology of the NPs. An FE-SEM image of CuO NPs at different magnifications can be seen in Figure 5A–D. The CuO NPs were regular in shape with a particle-like structure. CuO NPs have particle sizes ranging from 25.0 to 55.0 nm and uniform distributions. Images show some particles with square shapes and clusters that have agglomerated together. Stabilized NPs can also form clusters of material-like particles relatively close together. The NPs were stabilized and reduced by the peel extract, allowing them to be re-dispersed [48]. A peel extract limits clustering and flocculation in order to control particle size distribution. In order to fabricate nanoparticles within the ranges of small sizes, apple peel extract was found to be an effective stabilizing agent. As a result, the assembly of NPs was found by processes of aggregation, growth of particles, and also impurity adsorption [49]. EDX results of the synthesized NPs confirm their chemical composition and particle distribution of NPs on the whole surface. The existence of Cu and O in the NPs is demonstrated in Figure 5E. Thus, the only two components of synthesized NPs were copper and oxygen. The pattern made it clear that the NPs are crystalline structures made of two elements, such as Cu and O. Solid and strong signals were observed around 0.92 keV, 8.05 keV, and 8.91 keV for Cu with Cu La, Cu Ka, and Cu Kb representations, respectively, and 0.53 keV for O with O Ka representation [50,51]. In Figure 5G,H, the elemental map of CuO NPs shows homogeneous distributions of Cu and O, respectively. It was confirmed by EDX elemental analysis that Cu and O were present in a single particle, with an atomic percent composition of Cu at 64.19% and O at 35.81%. Due to the coating of the carbon with the NPs to measure the SEM analysis, it was not considered for the composition of elements present in the NPs. It was confirmed by these results that a CuO structure can be formed within five minutes through microwave synthesis.

### 3.6. Analysis of Bio-Reduced CuO NPs by HR-TEM

**Figure 6:**
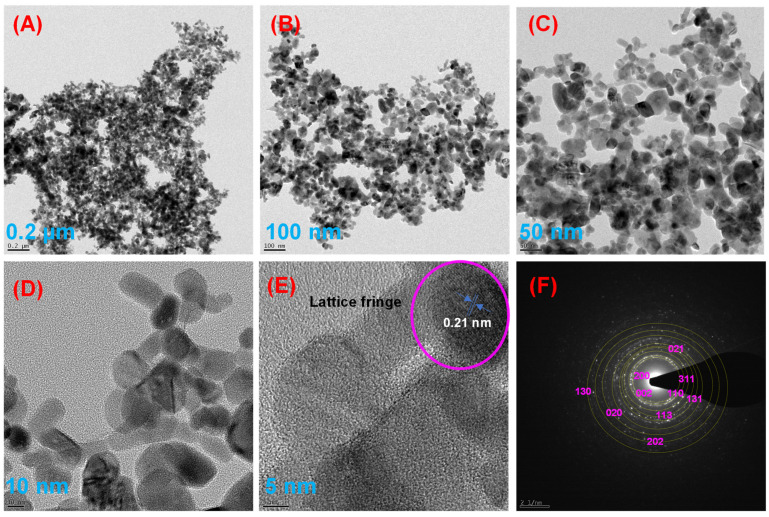
HR-TEM images of bio-reduced CuO NPs (**A**–**E**); SAED patterns of bio-reduced CuO NPs (**F**).

Figure 6A–E show HR-TEM images of the obtained CuO NPs with 31 nm scale bars. These TEM images and their SAED patterns indicate the size and crystallinity of the NPs [52,53]. These images show spherical NPs with an average diameter of 40.2 ± 4.0 nm and a narrow distribution of particle sizes. Analyses of particle size distributions were performed using ImageJ software. Due to NP agglomeration, spherical nature, and interconnections, Figure 6 closely matches SEM results. According to Figure 6F, the SAED pattern corresponds to the BCC crystalline structure of CuO, indexed to planes (021), (110), (002), (200), (130), (131), (202), (020), (002), (113), and (311). The spacing of the lattice fringes in one direction was about 0.21 nm. Morphological characterization of the NPs using FE-SEM and HR-TEM revealed that they were agglomerated.

### 3.7. Analysis of Bio-Reduced CuO NPs by XPS

**Figure 7:**
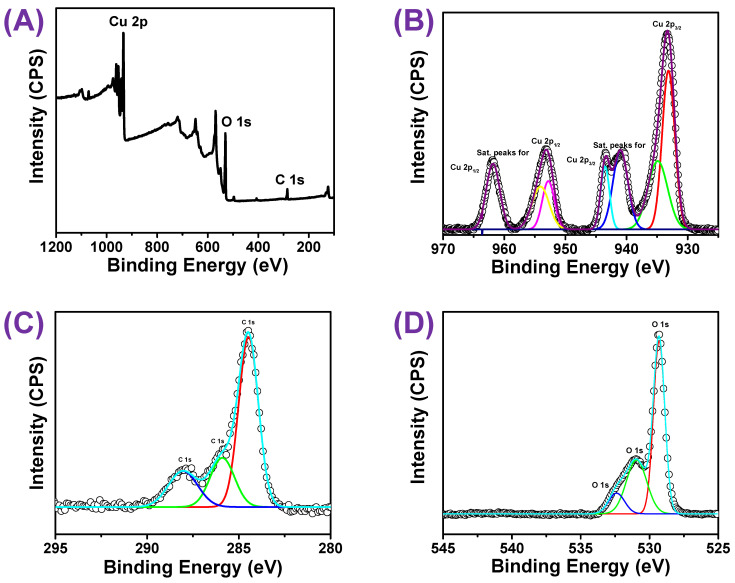
XPS of (**A**) survey scan spectrum, (**B**) Cu 2p, (**C**) C 1s, and (**D**) O 1s.

CuO NPs were analyzed using XPS analysis—a powerful surface-sensitive technique for the determination of oxidation state as well as chemical composition in the NPs [54]. For the standardization of all binding energies, the C 1s peak that appeared at 284.60 eV was used as a reference. According to Figure 7A, the peaks of the XPS wide scan spectrum were associated with Cu, C, and O elements. According to Figure 7B–D, the XPS spectra of Cu 2p, C 1s, and O 1s were measured with high-resolution (core XPS) spectra. The narrow energy range spectra of Cu 2p demonstrated a predominant peak at the stronger binding side of Cu 2p_3/2_ and increased binding energy, indicating an unfilled Cu 3d_9_ shell. The presence of Cu^2+^ in the CuO sample [55] was further confirmed by the presence of an unfilled Cu 3d_9_ shell. Additionally, the peaks at 953.28 eV and 933.38 eV in the core level spectrum of Cu 2p (deconvolution of CuO NPs, Figure 7B) can be attributed to two possibilities, such as Cu 2p_3/2_ and Cu 2p_1/2_ of CuO NPs, respectively. A high-resolution spectrum of carbon (C 1s) is shown in Figure 7C, which confirms the reference peak at 284.48 eV and other higher energy peaks at 286.08 eV and 288.41 eV. As a charge reference for the XPS spectra on the surface of NPs, the three peaks of the C 1s spectrum were known as contamination of adventitious carbon. The first peak of C 1s had a binding energy of 284.67 eV, indicating adventitious carbon containing the C–C bond; the second peak of C 1s had a binding energy of 286.08 eV, indicating adventitious carbon containing the C-O-C bond; and the third peak of C 1s had a binding energy of 288.08 eV, indicating adventitious carbon containing O–C=O bonds. According to the Gaussian–Lorentzian fit of O1s, two components were present at 529.08 eV and 530.88 eV (Figure 7D). This peak at 529.08 eV was attributed to the binding energy of lattice oxygen (O_L_)^2−^ in CuO lattices and agrees with O^2−^ in metal oxides (Cu^2+^ − O^2−^) [56]. It can be determined that the second peak at 530.88 eV reflects the binding energy for the oxygen vacancies or defects within the environment of CuO NPs [57]. According to the XPS spectra of NPs, there was no possibility of residual nitrogen in the precursor. As a result of measuring the XPS spectrum, CuO NPs were verified to be structurally stable.

### 3.8. Analysis of Bio-Reduced CuO NPs by TG/DTA

**Figure 8:**
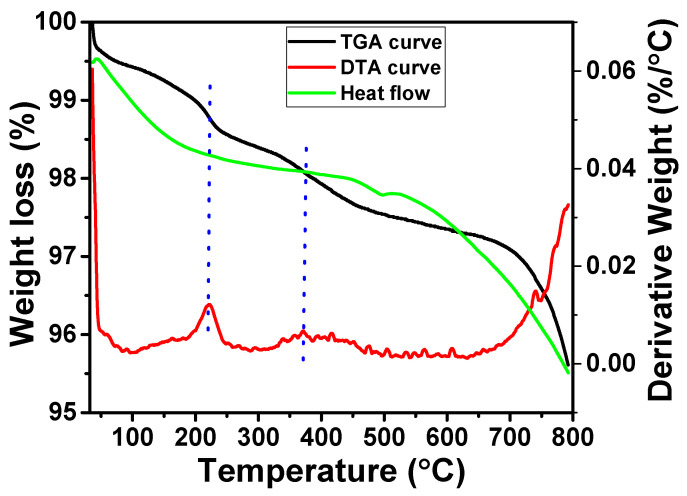
TGDTA curves of bio-reduced CuO NPs.

The thermal stability of the NPs was typically evaluated using the TGDTA curves, and a weight loss (in%) can be determined in relation to an increase in temperature [58]. The TGA and DTA curves of CuO NPs are displayed in Figure 8. The exothermic peak in the DTA curves at 201.9 °C indicated the release of energy from the surface of NPs into the surrounding environment. There was not much weight loss observed in the ranges of 30.0–800.0 °C [59]. However, the weight loss percentage was very little at three stages of temperatures, 110.0–210.0 °C, 300.0–410.0 °C, and 670.0–800.0 °C. The above three weight stages were mainly due to the release of moisture content and peel extract from the surface of NPs, as desorption had taken place during the thermal analysis [60]. Thus, the NPs were very stable up to 800.0 °C without decomposition.

### 3.9. Analysis of Bio-Reduced CuO NPs by BET Surface Area

**Figure 9:**
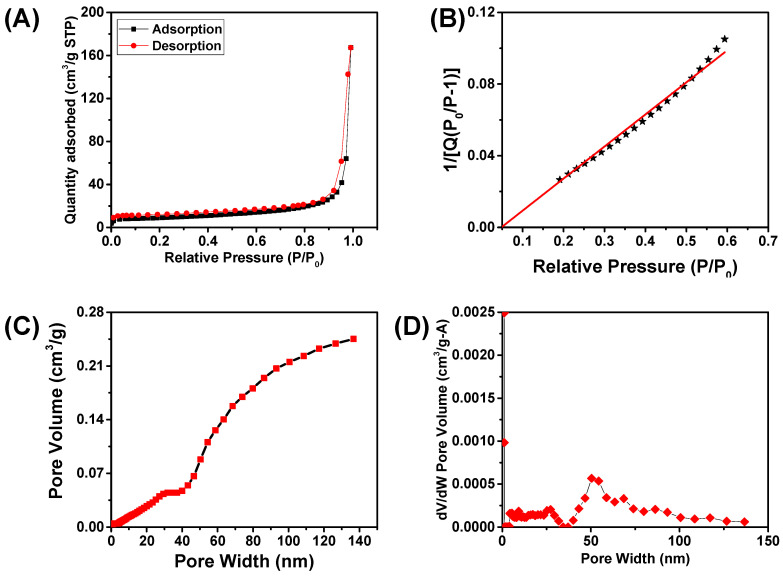
BET surface analysis with N_2_ gas adsorption–desorption isotherms of bio-reduced CuO NPs (**A**), surface area plot (**B**), BJH desorption pore size distribution (**C**), and differential pore volume plot (**D**).

The pore size distribution and surface area of CuO NPs were studied using N_2_ gas adsorption with BET surface area analysis. The adsorption–desorption curve follows a type IV isotherm obtained for the CuO NPs and is shown in Figure 9A. The hysteresis loop within the relative pressure (P/P_0_) ranges from 0.7 to 0.9 confirmed the presence of the mesoporous nature of the obtained NPs [61]. The surface areas were calculated to be 31.8240 m²/g by the standard multi-point BET (Figure 9B). The pore-size distribution was investigated for the obtained NPs by the desorption of the BJH method [62,63]. The pore size value was found to be 39.19 nm, which was clearly shown in Figure 9C, D. The results of pore size and surface area are consolidated in Table 1. Thus, the pore size shows that the NPs are mesoporous in nature which matched well with the porosity results obtained from the XRD analysis, FESEM, and also HRTEM images.

### 3.10. A Probable Mechanism of Bio-Reduced CuO NPs

**Scheme 2:**
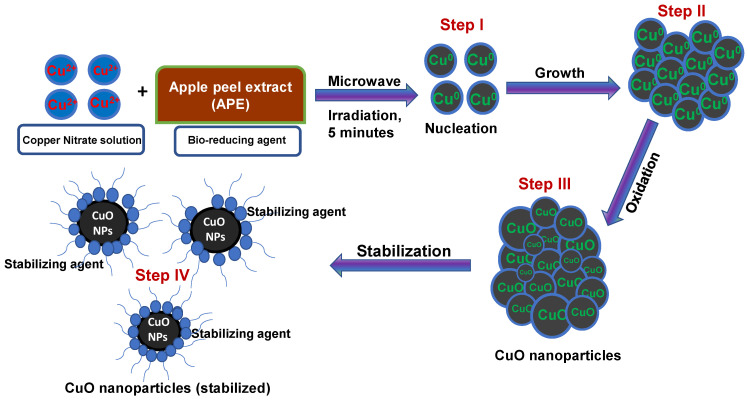
Schematic procedure for the bio-reduced CuO NPs [62].

An outline of the suggested CuO NPs mechanism is given in Scheme 2. As part of the biosynthetic process, there were usually three processing stages, which include the activation, growth, and termination phases [64,65]. In this process, the extract may act as a reducing as well as a stabilizing agent. In an initial step, a CuNO_3_·2H_2_O salt precursor would be dissolved in distilled water and activated by removing its cations. Copper ions and AP extract undergo an oxidation–reduction reaction during mixing. Cationic copper was reduced and the oxidation state became 2+ into a metallic form. The nucleation process (Cu^0^) involves the direct conversion of electron-rich natural constituents (AP extract as a bio-reducing agent) directly into CuO NPs due to the greater chemical reactivity on their surfaces. The hydroxyl group of the AP extract effectively participated in the reducing process, and as evident in Figure 2, a broad peak is visible at 3416.0 cm^−1^ for the hydroxyl groups in the AP extract. During the oxidation process, the Cu^0^ was progressively combined and the growth of CuO NPs commenced [66]. In the third stage, CuO NPs were stabilized. A strong protective shield layer formed and it surrounded the entire surface of the nucleated NPs; hence, limiting the growth of NPs. Additionally, these extracts use steric pressures to keep the capped NPs apart [67]. Similarly, food waste materials contain a variety of bioactive components that could aid in lowering metal ions or metal oxides as well as stabilizing metallic NPs [68].

### 3.11. Antibacterial Efficacy of Bio-Reduced CuO NPs

**Figure 10:**
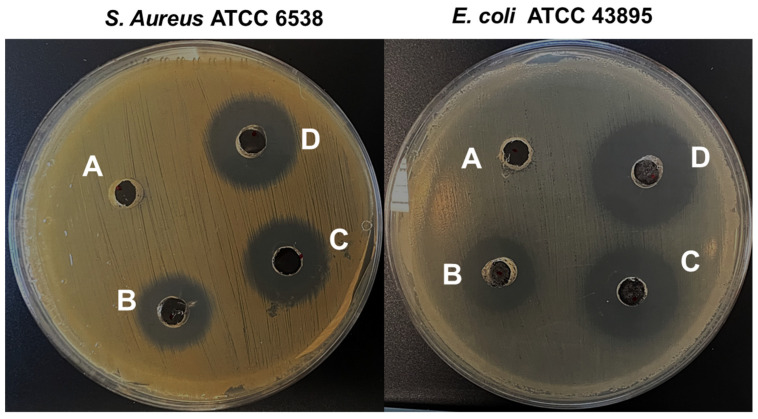
Antibacterial efficacy of bio-reduced CuO NPs with different concentrations (**A**, 0 µg/mL; **B**, 100 µg/mL; **C**, 200 µg/mL; and **D**, 300 µg/mL).

Both Gram-positive and Gram-negative bacteria tested were susceptible to CuO NPs. *E. coli* and *S. aureus* were susceptible to NPs with MIC values of 50.0 μg/mL and MBC 100 μg/mL, respectively. Additionally, the agar diffusion test was carried out for the assessment of the antibacterial activity of CuO NPs and showed a clear zone for the activity against both bacteria. The diameters of inhibition zones after the treatment are consolidated in Table 2 and representative images are shown in Figure 10. The results revealed that the zone of inhibition grew significantly with respect to the concentration of CuO NPs. There was a direct relationship between the zone of inhibition and the concentrations of NPs. In *E. coli* and *S. aureus*, the zone of inhibition was determined to be 29.0 ± 2.3 mm and 26.0 ± 1.1 mm, respectively. CuO NPs have been shown to be particularly effective against various types of bacterial strains [69]. The highly unique surface characteristics of CuO NPs to volume ratio permit them to interact across the surface of the cell membrane of the bacterial pathogen, finally killing the bacterial pathogen [70]. Particularly, electronic interactions, produced by CuO NPs with a smaller size and a larger surface area, were helpful to enhance the surface responsiveness of NPs. Additionally, the improved surface area percent instantly acts together with the bacterium, causing enhanced bacteria interaction during the process. These two crucial elements (Cu and O in NPs) play a significant role in enhancing the antibacterial efficacy of NPs with a large surface area [71].

### 3.12. Antibiofilm Potency of Bio-Reduced CuO NPs against E. coli and S. aureus

**Figure 11:**
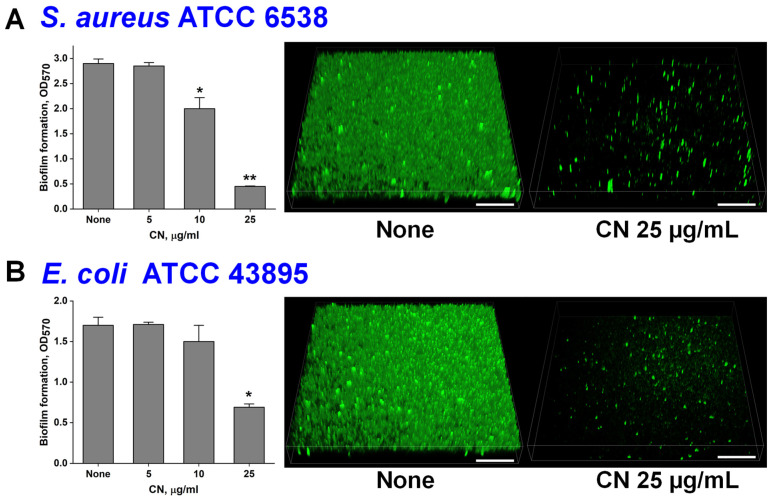
Effects of bio-reduced CuO NPs on *S. aureus* (**A**) and *E. coli* (**B**) biofilm formation. ** *p* < 0.01 * *p* < 0.05 Vs. non-treated controls. Scale bars represent 100.0 μm.

A biofilm potency of NPs was performed against *E. coli* and *S. aureus*. Figure 11 shows the dose-dependent antibiofilm inhibition after the treatment with NPs at doses of 5.0 µg/mL, 10.0 µg/mL, and 25.0 µg/mL. As a result, NPs at a lower dose of 10.0 µg/mL inhibited 12.0 ± 10.7 and 31.0 ± 7.2% biofilm formation by *E. coli* and *S. aureus* after 24 h of incubation, respectively. Furthermore, when the dose of NPs was increased to 25.0 µg/mL, significant inhibition of *E. coli* and *S. aureus* biofilm formation was observed >60.0 ± 2.3 and 80.0 ± 0.3%, respectively. The CuO-containing polymeric or non-polymeric NPs were reported to be antibacterial as well as antibiofilm (28.0 to 69.0%) agents against *E. coli* and *S. aureus* [69], *Bacillus subtilis*, and *Pseudomonas aeruginosa* [72]. In the current study, the synthesized NPs inhibited selective *E. coli* and *S. aureus* biofilm at lower concentrations, whereas as concentration increased the NPs showed potential antibacterial efficacy against both tested bacterial strains. Additionally, reduced biofilm thickness was confirmed by confocal laser microscopy (Figure 11) and COMSTAT biofilm analysis (Table 3). NPs at 25.0 µg/mL reduced biomass and mean thickness of both pathogens, *E. coli* and *S. aureus*, by >65.0 and 85.0%, respectively, according to untreated controls (Table 3). From an applications point of view, CuO NPs are used in various industry sectors; moreover, there is a medical application of CuO NPs as antibacterial material [73]. Concerns are raised by their toxicity, which includes toxicity to the blood and immune system, but knowledge of their immunotoxicity is still relatively restricted. CuO NPs or material decorated with NPs prevents the growth of biofilm or adherent populations of microorganisms on the surface of materials [74]. Recently, Boliang et al. reported in 2022 that biosynthesized CuO NPs enhance antibiofilm activity against *K. pneumonia* and *S. aureus* [75].

## 4. Conclusions

For the quick one-pot production of CuO NPs, pressure control or a higher temperature was not necessary to maintain. The XRD pattern revealed that all of the NPs had a monoclinic crystalline structure. According to XPS and EDX studies, Cu and O make up the NPS. The pore-size distribution was investigated for the obtained NPs by the desorption of the BJH method, which revealed that the NPs are mesoporous in nature. The pore size for the NPs was found to be 39.19 nm. An in vitro antibacterial efficacy of CuO NPs was tested against the bacterial pathogens *E. coli* and *S. aureus.* The results revealed a clear zone of inhibition against both bacterial strains. The highly unique surface characteristics of CuO NPs to volume ratio enable them to interact across the surface of the bacterial cell membrane eventually leads to killing them. A biofilm assay was also performed to examine the antibiofilm potency of NPs against both pathogens. NPs at 25.0 µg/mL reduced the biomass and mean thickness of both the bacterial pathogens by >85.0% and 65.0%, with respect to the untreated controls. In the near future, disease detection may be the primary focus of CuO NPs’ biological uses; however, they may also have potential applications in a wide range of other fields.

## Figures and Tables

**Table 1:** Surface area, pore volume, and pore size distribution of CuO NPs by BET analysis.

Material	Surface Area (m²/g)	Pore Volume (m²/g)			Pore Size (nm)	
		t-Plot Micropore Volume	BJH Adsorption Cumulative Volume of Pores	BJH Desorption Cumulative Volume of Pores	BJH Adsorption Average Pore Diameter (4V/A)	BJH Desorption Average Pore Diameter (4V/A)
CuO NPs	31.8240	0.005294	0.255223	0.251015	42. 44	39.19

**Table 2:** Antibacterial efficacy of bio-reduced CuO NPs at different concentrations against *S. aureus* and *E. coli* by a zone of inhibition.

Name of the Bacterial Strains	Zone of Inhibition (mm)			
	Conc. of CuO NPs (µg/mL)			
	300.0	200.0	100.0	0
*S. aureus*	26.0 ± 1.1	23.0 ± 1.3	19.0 ± 0.9	ND
*E. coli*	29.0 ± 2.3	26.0 ± 0.8	20.0 ± 0.5	ND

**Table 3:** COMSTAT analysis of biofilm biomass, mean thickness, and substratum coverage spatial characteristics.

Bacterial Strains	Biofilm Biomass (µm^3^ µm^−2^)		Mean Thickness (µm)		Substratum Coverages (%)	
	None	25.0 µg/mL	None	25.0 µg/mL	None	25.0 µg/mL
*S. aureus*	43.02 ± 2.4	6.11 ± 0.6	46.38 ± 3.1	11.36 ± 1.1	100 ± 0.8	8.01 ± 1.2
*E. coli*	40.80 ± 1.9	9.01 ± 1.0	39.42 ± 1.1	16.40 ± 0.4	100 ± 0.4	12.4 ± 2.1

## Data Availability

The datasets used or analyzed during the current study are available from the corresponding author upon reasonable request.

## Supplementary Materials

The following supporting information can be downloaded at: https://www.mdpi.com/article/10.3390/ma16010217/s1; the preparation of extract, biofilm observations by confocal laser scanning microscopy, and instruments used for the characterization of CuO NPs are provided in the Supplementary Materials.
