# Supplementary material for: One Pot Synthesis of Copper Oxide Nanoparticles for Efficient Antibacterial Activity
Source: Materials (Basel). 2022 Dec 26;16(1):217. doi: 10.3390/ma16010217 (PMC9822411; doi:10.3390/ma16010217)
Supplement: Supplementary file 1 [file materials-16-00217-s001.zip › materials-2087942-supplementary.pdf]

Microwave-Assisted Synthesis of Copper Oxide Nanoparticles by Apple Peel Extract and Their Efficient Antimicrobial Activity

Rajaram Rajamohan* Chaitanya Jayprakash Raorane, Seong-Cheol Kim*, Yong Rok Lee*

School of Chemical Engineering, Yeungnam University, Gyeongson 38541, Republic of Korea

* Correspondence: rajmohanau@yu.ac.kr (R.R); sckim07@ynu.ac.kr (S.C.K); yrlee@yu.ac.kr (Y.R.L)

Preparation of Apple peel extract

A peeler was used to get a peel of fresh red delicious apples with one-kilogram weight. A chilled waring blender was used to homogenize the obtained apple peels (100.0 mg, 10.3% of the fresh apple weight) for 5 minutes with cool 80.0 % acetone (1:2, w/v). For a further 3 minutes, samples were homogenized using a Polytron homogenizer. A vacuum at 45.0 °C was used to evaporate approximately 90.0 % of the filtrate. Following the first extraction step, the residue (100.0 mg, 10.2 % apple peels) was resuspended in 500.0 mL water, extracted thrice with ethyl acetate solvent, and also with water-saturated n-butanol.

Biofilm observations by Confocal laser scanning microscopy

For the CLSM assay, single-strain biofilms of both bacterial pathogens were produced in 96-well plates (presence and absence of CuO NPs) at 37.0 °C for 24 hours without shaking. The free-floating cells were then discarded by rinsing with distilled water twice, and biofilm cells were attached to the surface of the wells that were stained with CFDA-SE (Make: Invitrogen, Model: Molecular Probes, Inc, Place: Eugene, Country: USA). The bottom of each well was then visualized using a 488.0 nm Ar laser (emission ranges 500.0 - 550.0 nm) using a CLSM (Model: Nikon, Place: Tokyo, Country: Japan). COMSTAT software to calculate the

mean biofilm thicknesses (μm), biomass ($\mu m^3/\mu m^2$), and substratum coverages (%). There were two independent samples analyzed for the individual experiment, and greater than 5 random spots were detected.

Instruments Used

Various techniques were used to analyze the NPs, including diffusion reflectance spectroscopy, FT-IR spectroscopy, Raman spectroscopy, FE-SEM with EDX spectroscopy, HR-TEM, XRD, XPS, TGDTA, and BET surface area analysis. DRS is measured using an OPTIZEN 3220 UV spectrophotometer within the wavelength ranges of 200.0 - 800.0 nm. FT-IR spectra are recorded with the Perkin Elmer Spectrum Two in transmittance mode within the ranges of 400.0 - 4000.0 cm^{-1}. 16 scans for the measurement at a resolution of 8 cm^{-1}. Raman spectral measurements were captured on the XploRA Micro-Raman spectrophotometer (Horiba) within the spectral shift ranges of 100.0 - 1500.0 cm^{-1}. At a 10.0 kV accelerating voltage, FE-SEM, and EDX spectral analysis was carried out with the Hitachi S-4800. HR-TEM images are captured using an FEI-Tecnai TF-20 with a 120.0 kV operating accelerating voltage. Powder XRD measurements were performed on a PANalytical X'Pert3 MRD diffractometer at 40.0 kV and 30 mA with monochromatized Cu K radiation (λ =1.54 Å). The 2θ range is 10° - 80° at a scan rate of 5° min^{-1} and a wavelength of 1.5405 Å. K-Alpha is used to generate XPS spectra (Thermo Scientific). The CasaXPS software S3 is used to deconvolve the high-resolution XPS spectra. The thermal behavior of the NPs is performed using TA instruments, and the curves are of individual elements analyzed using the Universal V4.5A Program. The weight of each sample is around 5.5 mg and the temperature range for the measurement is about 35.0 - 700.0 °C with increments of 10 °C/minute. All of the above instrument services were utilized at the core research support center (CRSC) for natural products and medical materials at Yeungnam University, South Korea.
